# Exome‐wide association of deltamethrin resistance in *Aedes aegypti* from Mexico

**DOI:** 10.1111/imb.12575

**Published:** 2019-03-13

**Authors:** K. Saavedra‐Rodriguez, C. L. Campbell, A. Lenhart, P. Penilla, S. Lozano‐Fuentes, W. C. Black

**Affiliations:** ^1^ Department of Microbiology, Immunology and Pathology Colorado State University Fort Collins CO USA; ^2^ Division of Parasitic Diseases and Malaria Center for Global Health, Centers for Disease Control and Prevention Atlanta GA USA

**Keywords:** *Aedes aegypti*, SNP association, deltamethrin resistance

## Abstract

*Aedes aegypti* is the major vector of a number of arboviruses that cause disease in humans. Without vaccines or pharmaceuticals, pyrethroid insecticides remain the major tool for public health protection. Pyrethroid resistance is now widespread. Replacement substitutions in the voltage‐gated sodium channel (*vgsc*) that reduce the stability of pyrethroid binding account for most of the resistance, but metabolic mechanisms also inactivate pyrethroids. High‐throughput sequencing and the *A. aegypti* L5 annotated physical map has allowed interrogation of the exome for genes and single‐nucleotide polymorphisms associated with pyrethroid resistance. We exposed females of *A. aegypti* from Mexico to a deltamethrin discriminating dose to designate them as resistant (active after 1 h) or susceptible (knocked down with no recovery after 4 h). The *vgsc* on chromosome 3 had the highest association, followed by genes proximal to *vgsc*. We identified potential detoxification genes located singly (eg HPX8C) or within clusters in chromosome 2 [three esterase clusters, two of cytochrome P450 monooxygenases (CYP)] and chromosome 3 (one cluster of 16 CYP325 and seven CYP9 genes). Deltamethrin resistance in *A. aegypti* is associated with mutations in the *vgsc* gene and a large assortment of genes.

## Introduction

Insecticide resistance in *Aedes aegypti* mosquito populations threatens our ability to control arboviral diseases such as dengue, chikungunya, and Zika. During the last decade, intense global reliance on pyrethroid insecticides in vector control programmes worldwide has resulted in widespread pyrethroid resistance in *A. aegypti* populations (da‐Cunha *et al.*, 2005; Harris *et al.*, 2010; Marcombe *et al.*, 2012; Aponte *et al.*, 2013; Flores *et al.*, 2013). Assessment and characterization of pyrethroid resistance mechanisms are important to the establishment of insecticide resistance management plans as insecticidal spraying efforts increase.

Various mechanisms of resistance have been reported in insects, including the insensitivity of the target site, increased metabolism, reduced penetration throughout the cuticle and changes in behaviour that usually result in avoidance of insecticide exposure. Although insecticide resistance is multifactorial, most research has focused on target site insensitivity and metabolic mechanisms separately, whereas reduced penetration and behavioural changes are just starting to be understood (Zalucki and Furlong, 2017; Balabanidou *et al.*, 2018).

Pyrethroids are neurotoxins that target the voltage‐gated sodium channel (*vgsc*) in insect neurons, resulting in a disrupted action potential that culminates with rapid knockdown and mortality. Specific amino acid substitutions at *vgsc* result in target insensitivity in resistant insects. Around 57 nonsynonymous mutations in the *vgsc* have been associated with channel insensitivity (Rinkevich *et al.*, 2013); interestingly, convergence of knockdown resistance (kdr) mutations across different insect genera validate the important role of this mechanism. In *A. aegypti*, 11 nonsynonymous substitutions from different areas of the world have been reported (Rinkevich *et al.*, 2013; Saavedra‐Rodriguez *et al.*, 2018). Currently, five have been confirmed to contribute to insecticide resistance by electrophysiology (Du *et al.*, 2013; Hirata *et al.*, 2014; Haddi *et al.*, 2017). Interestingly, co‐existence of kdr mutations at different loci is common in insect species; furthermore, in *A. aegypti* these kdr mutations seem to be geographically distributed, conferring different levels of resistance (Hirata *et al.*, 2014; Saavedra‐Rodriguez *et al.*, 2018). The three loci examined in this paper are 410, 1016 and 1534 corresponding to the position of amino acid substitutions. A valine at locus 410 (V410) confers susceptibility, whereas leucine (L410) confers resistance. A valine at locus 1016 (V1016) confers susceptibility, whereas isoleucine (I1016) confers resistance. A phenylalanine at locus 1534 (F1534) confers susceptibility, whereas cysteine (C1534) confers resistance.

Increased metabolism can result from overexpression (by amplification or cis–trans regulation) of insecticide metabolizing enzymes or by enzyme variants that provide higher or insecticide‐specific catalytic properties (Hemingway and Karunaratne, 1998). Four enzyme families are associated with insecticide metabolism: cytochrome P450 monooxygenases (CYP), carboxyl/choline esterases (CCE), glutathione‐*S*‐transferases (GSTs) and reduction/oxidation (Red/ox) enzymes. Identification of detoxification genes in *A. aegypti* confirm the high redundancy of genes potentially involved in insecticide metabolism, describing 160 CYP, 44 CCE and 26 GSTs (Strode *et al.*, 2008). With improved microarray technology, whole transcriptomes had been interrogated to identify candidate genes conferring insecticide resistance in field or in artificially selected *A. aegypti* strains (Strode *et al.*, 2008; Marcombe *et al.*, 2009a; Bingham *et al.*, 2011; Bariami *et al.*, 2012; Grisales *et al.*, 2013; Kasai *et al.*, 2014). Unsurprisingly, genes and their expression patterns varied among regions of the globe. Nonetheless, a few common genes were successfully confirmed to produce insecticide‐metabolizing proteins (Stevenson *et al.*, 2012).

As next‐generation sequencing becomes more affordable, whole genomes or transcriptomes are interrogated for association with specific phenotypic traits. Few studies using this technology in mosquitoes have assessed the transcriptional changes and replacements identified in permethrin‐resistant *A. aegypti* (David *et al.*, 2014; Faucon *et al.*, 2015). A recent complete partially annotated physical map of the *A. aegypti* genome (Matthews *et al.*, 2018) will allow us for the first time to perform genome‐wide association studies. In the present study, we use an exome‐enrichment genomic DNA sequencing approach (Juneja *et al.*, 2015) to identify single‐nucleotide polymorphisms (SNPs) associated with deltamethrin resistance. We analysed an *A. aegypti* collection from Viva Caucel from Yucatán State in southern Mexico that was subjected to permethrin selection in the field from 1998 to 2010. Our hypothesis was that replacements in detoxification genes that have significantly different frequencies between resistant and susceptible mosquitoes are indicative of their potential involvement in deltamethrin resistance or are due to their physical proximity to *vgsc* on chromosome 3. The expected *vgsc* replacement polymorphisms were identified in the dataset and also allowed us to identify genome regions surrounding *vgsc* that are likely to have been involved in a genetic sweep.

## Results

### Resistance phenotype

**Figure 1:**
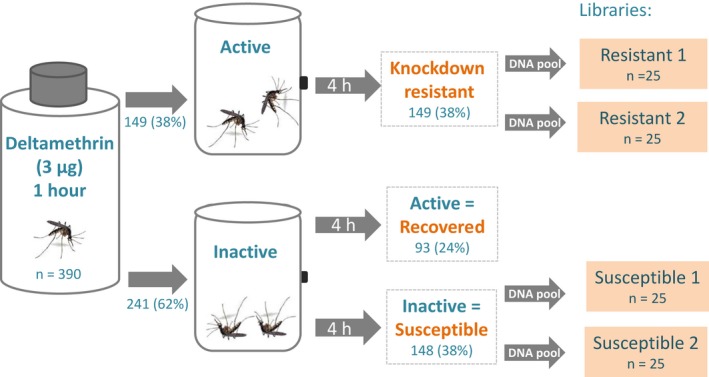
Procedure to discriminate phenotypes and to generate pools of deltamethrin‐resistant and susceptible *Aedes aegypti*.

Deltamethrin resistance was phenotyped by exposing 390 *A. aegypti* adults from a field resistant strain collected in Viva Caucel to bottles coated with 3.0 µg of deltamethrin during 1 h. This concentration discriminated three phenotypes: (1) 149 mosquitoes were ‘knockdown resistant’ (38%), (2) 148 were ‘susceptible’ (38%) and (3) 93 ‘recovered’ (24%) (Fig. 1). In this study, we compared pooled mosquitoes from the ‘knockdown resistant’ and ‘susceptible’ groups (Fig. 1). We excluded the ‘recovered’ group to remove the uncertainty of assigning the incorrect phenotype. Also, we assumed that more contrasting phenotypes would allow us to get more contrasting SNPs underlying deltamethrin resistance.

### Exome sequencing

We obtained between 39 million and 43 million sites in each of the four libraries. These libraries were (1) alive mosquitoes replicate 1 (VCD_1A), (2) alive mosquitoes replicate 2 (VCD_2A), (3) dead mosquitoes replicate 1 (VCD_1D) and (4) dead mosquitoes replicate 2 (VCD_2D). There were 39 830 841 sites in VCD_1A, 43 502 656 reads in VCD_2A, 42 819 797 in VCD_1D and 43 255 535 in VCD_2D. After removing repetitive DNA (coverage >1000) and sites with less than 25 reads, we finished with (9–14) × 10^6^ reads per library to be analysed. We performed a heterogeneity *χ* ^2^ test to select sites sharing similar coverage within replicates in the knockdown‐resistant (8 998 195) or susceptible libraries (8 652 414). From these sites, we identified 7 055 061 common sites between knockdown‐resistant and susceptible groups (this represents 26% of the targeted exome) (Table S1). Around 6.4% of the sites were polymorphic (454 380) and subject to a contingency *χ* ^2^ analysis and then assigned a genetic association value −log_10_(prob). We acknowledge that by limiting our analysis to 26% we might have missed causal resistance sites.

**Figure 2:**
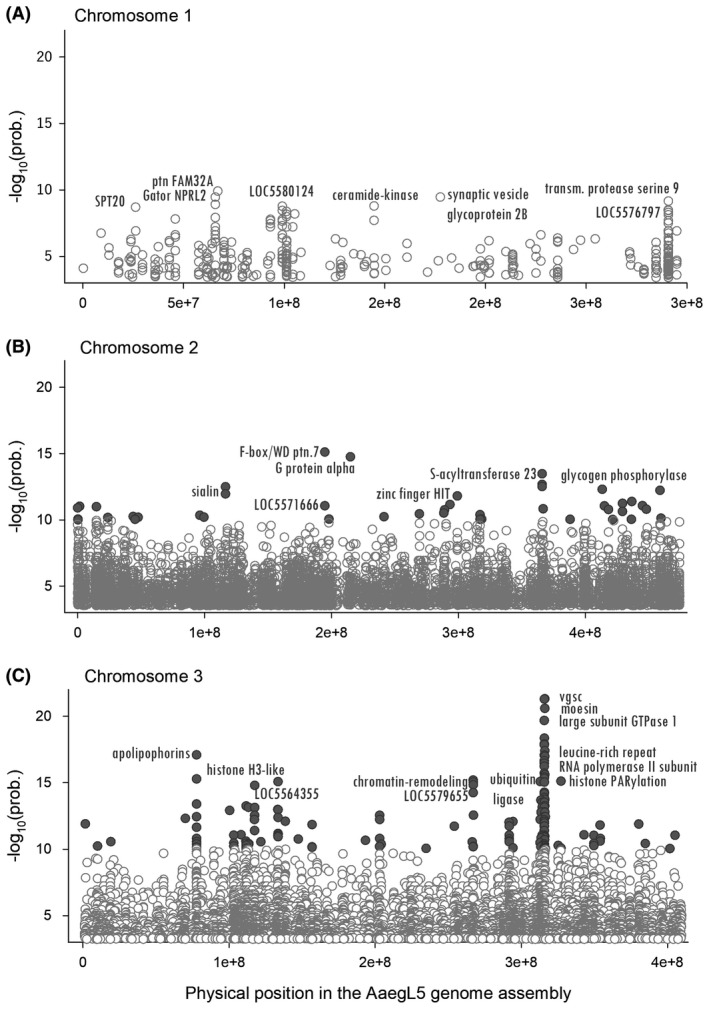
Significant SNPs differing between knockdown resistant and susceptible *Aedes aegypti* at chromosomes 1, 2 and 3. Only single‐nucleotide polymorphisms surpassing the false discovery rate threshold are shown, including synonymous and nonsynonymous substitutions, introns, 3′‐untranslated region (UTR), 5′‐UTR regions and noncoding RNAs.

A Benjamini–Hochberg correction (Benjamini and Hochberg, 1995) for false discovery rate (FDR) was applied to chromosomes 1, 2 and 3 separately (*α* = 0.01). A total of 14 532 SNPs (0.206% of the targeted exome) surpassed the FDR thresholds of 3.39, 3.52 and 3.22 for chromosomes 1, 2 and 3 respectively. These SNPs occurred in the 3′‐untranslated region (UTR; *n* = 321), 5′‐UTR (*n* = 443), coding sequence (*n* = 10 359; of which 2614 were nonsynonymous and 7745 were synonymous substitutions), intergenic regions (*n* = 40), noncoding RNA (*n* = 118) and introns (*n* = 3251). Fig. 2 shows the distribution of SNPs with −log_10_(prob) values above the FDR along chromosomes 1, 2 and 3. We provide a complete list of significant SNPs in Tables S2, S3 and S4. The approach used in this paper assumes that significant SNPs are independently distributed (in linkage equilibrium). This assumption is invalid, but we have no understanding of linkage disequilibrium blocks or haplotype structure in this or other mosquito species. Instead, we focus our results and discussion on those genes with the largest −log10(prob) values.

### Highest associations occur at the target site (*vgsc*) and surrounding genes

From 14 532 significant SNPs across the exome, 423 were in chromosome 1, 7150 in chromosome 2 and 6959 in chromosome 3 (Fig. 2). From these SNPs, high association values [−log_10_(prob) > 10] were identified primarily in chromosome 3 (168 SNPs) and secondly in chromosome 2 (54 SNPs). The highest −log_10_(prob) in the whole exome [−log_10_(prob) > 20] occurred specifically at *vgsc* (LOC5567355) and surrounding genes (moesin and large GTPase subunit). From 52 to 80% of *vgsc* was covered across libraries (out of 7794 nucleotides covered by enrichment probes covering exons 5–30 and introns 4–29) and 19 SNPs were significantly associated with deltamethrin resistance. Notably, only nonsynonymous mutation V410L was highly associated with deltamethrin.

In *vgsc*, 10 out of the 19 significant SNPs had −log_10_(prob) > 10, including eight SNPs located at introns 4, 6, 7 and 8, nonsynonymous substitution at V408L (V410L based on *Musca domestica* sodium channel numbering accession no. X96668) and a synonymous substitution at D375 in exon 8 (D380 in *M. domestica*). The remaining SNPs in *vgsc* [−log_10_(prob) < 10) occurred at introns and one nonsynonymous substitution at S679T. Interestingly, S679T corresponds to residue V723 in *M. domestica*, probably located at the linker IS6–IIS1 in the *vgsc*. Mutations at the IS6–IIS1 linker are unusual; only C785R has been identified in pyrethroid‐resistant *Blatella germanica* in combination with E435K+L1014F (Rinkevich *et al.*, 2013).

**Figure 3:**
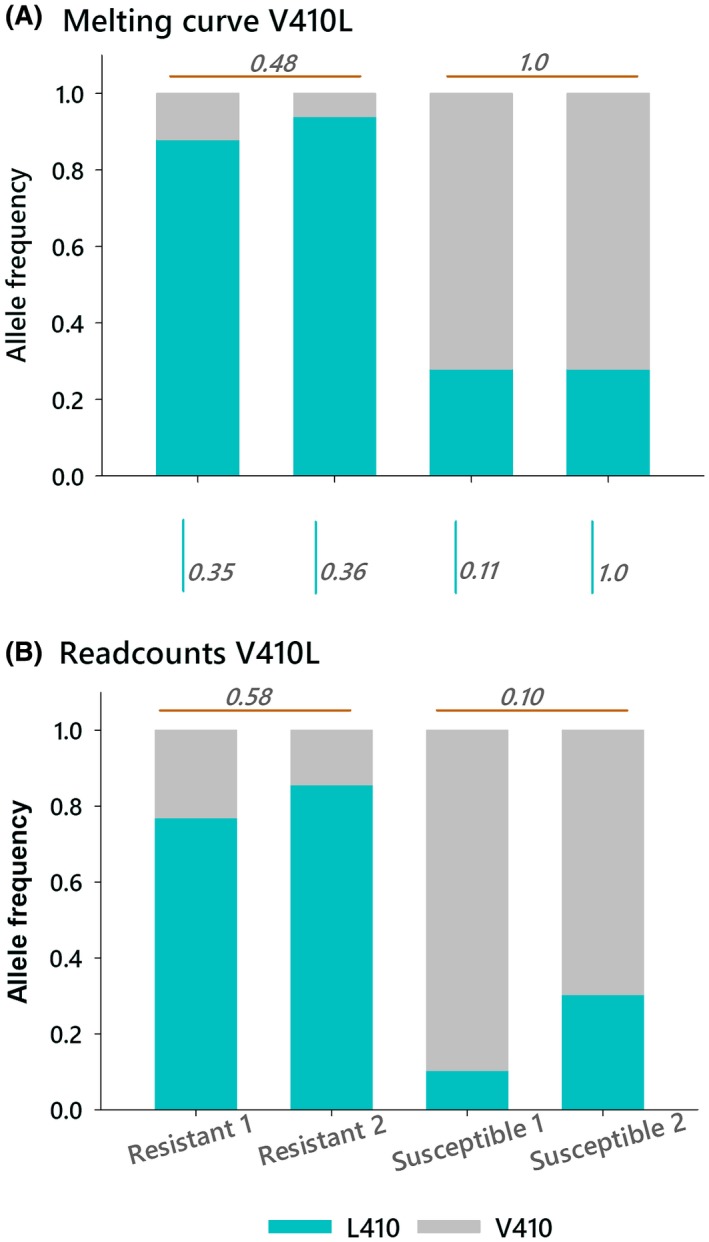
Allele frequencies of knockdown resistance mutation V410L obtained by (A) individual genotyping (melting curve assay) and by (B) library read counts. Lines above each bar represent pairwise comparisons between replicates, and the numbers on each line indicate the probability from the Fisher's exact test. The numbers and lines between graphs indicate the probabilities from the Fisher's exact tests between techniques.

Our results showed that V410L located at IIS6 in *vgsc* had a high association [−log_10_(prob) = 17] with deltamethrin resistance. This mutation was recently associated with pyrethroid resistance in *A. aegypti* (Haddi *et al.*, 2017; Saavedra‐Rodriguez *et al.*, 2018). To determine whether the library sequences accurately estimated the population of V410L allele frequencies, we performed a melting curve PCR SNP assay to genotype the individuals used to construct the four libraries (Saavedra‐Rodriguez *et al.*, 2018). Fig. 3 shows the comparison of V410L allele frequencies obtained by library sequencing and by melting curve PCR SNP genotyping. Allele frequencies were not significantly different between replicates in any of the comparisons. Individual genotyping shows that the frequency of the resistant allele L410 was 0.88 and 0.94 in the knockdown‐resistant individuals used in the replicates and 0.28 for the susceptible individuals used in the replicates (Fig. 3). In the sequencing libraries, L410 had a −log_10_(prob) = 17; frequencies in the knockdown‐resistant libraries for replicates 1 and 2 were 0.88 and 0.73 respectively. In the susceptible individuals, frequencies were 0.10 and 0.3 in replicates 1 and 2 respectively. Heterogeneity *χ* ^2^ tests showed that the allele frequencies were similar regardless of the detection technique used, suggesting that the frequencies of the *vgsc* alleles in the sequencing libraries accurately estimated population frequencies.

### Testing for a genetic sweep by *vgsc*

**Figure 4:**
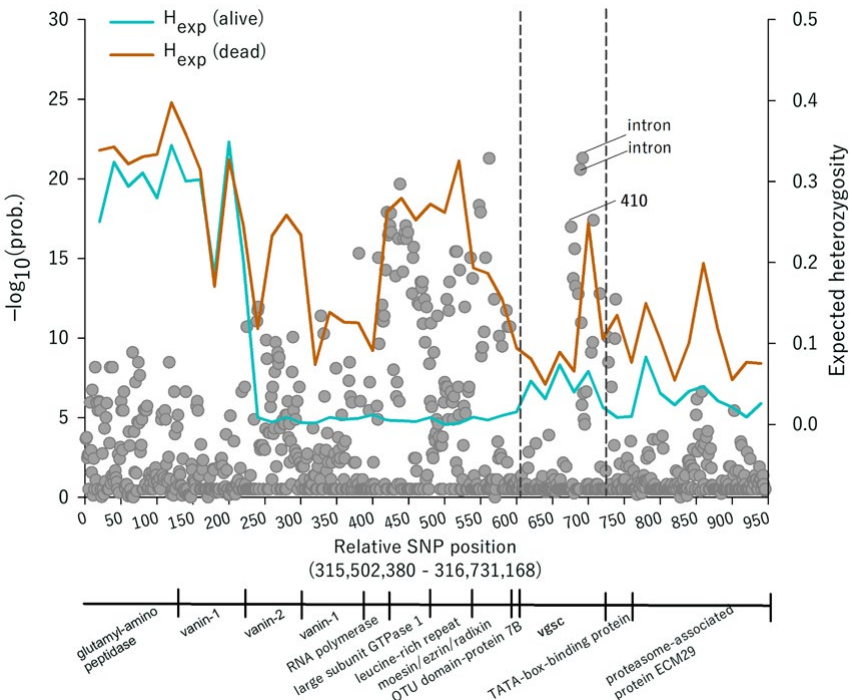
Signatures of a selective sweep by voltage‐gated sodium channel (*vgsc*). The lines show the expected heterozygosity *H*_exp_ for single‐nucleotide polymorphisms (SNPs) at *vgsc* and upstream and downstream genes. Dots show the individual SNP −log_10_(prob). The physical boundaries between genes are shown below the plot.

Because 45% of the 168 SNPs with high −log_10_(prob) > 10 were located in close proximity to *vgsc* (Fig. 2C), we analysed a 1.4 Mbp region upstream/downstream of this gene. By observing SNPs with −log_10_(prob) > 10 in this region, the sweep extended from a TATA‐box binding protein upstream to *vgsc* to a vanin‐2 protein downstream to *vgsc*. Genes in this sweep included a ubiquitin protease (OTU domain‐containing protein 7B), a cytoskeleton binding protein (moesin/ezrin/radixin), a protein that forms a structural framework for protein–protein interactions (leucine‐rich repeat‐containing protein 47), a ribosomal biogenesis protein (large subunit GTPase) and two vanins involved in nitrogen compound metabolic processes (Fig. 4). Since none of these protein‐coding genes have been functionally or genetically associated with insecticide resistance, we tested the possibility of a genetic sweep by rapid positive selection at *vgsc* rather than being selected due to their own activity as resistance loci. Fig. 4 shows the expected heterozygosity *H*_exp_ of SNPs at *vgsc* and surrounding genes in knockdown‐resistant and susceptible mosquitoes, where $H_{\exp}=1-\sum_{i=1}^{n}pi^{2}$ and *n* is the number of alternate nucleotides at a site. Heterozygosity at position L410 was low in knockdown‐resistant mosquitoes and high in knockdown‐susceptible mosquitoes. This is expected to occur when beneficial mutations (at *vgsc*) are rare; but once a beneficial mutation has occurred it increases in frequency rapidly, thereby drastically reducing genetic variation in the population. To determine whether this pattern was genome wide, we tested for this pattern throughout all three chromosomes. Table 1 and Fig. S1 display the result of three pairwise *t*‐tests, one for each chromosome testing whether the mean of the difference between heterozygosities in resistant and susceptible mosquitoes is different from zero. For each pairwise comparison of SNPs with −log_10_(prob) greater than the FDR we subtracted the expected heterozygosity of susceptible mosquitoes, Het (dead), from the heterozygosity of resistant mosquitoes, Het (alive). When Het (alive) > Het (dead) then the difference is positive, but when Het (dead) > Het (alive) the difference is negative. We would expect genes in surviving mosquitoes to have been swept and, therefore, have a lower heterozygosity. Conversely, we would expect genes in dead mosquitoes (homozygous susceptible) not to have been swept or swept at a lower rate (heterozygotes) and, therefore, have a higher heterozygosity. Along chromosomes 1 and 3 the mean difference was negative and significant in the paired *t*‐test (Table 1), consistent with genetic sweeps.

**Table 1:** Pairwise *t*‐tests comparing heterozygosities in alive versus dead mosquitoes. The mean is the average of Het (alive) −  Het (dead)

Chromosome	*N*	Mean	95% CL	Mean	*t*‐value	Pr > |*t*|	Sweep?
1	431	−0.103	−0.125	−0.082	−9.59	<.0001	Y
2	7414	0.038	0.032	0.043	14.03	<.0001	N
3	7132	−0.042	−0.048	−0.037	−14.74	<.0001	Y

However, along chromosome 2 the mean difference was positive and significant in the paired *t*‐test (Table 1). Most importantly, in all three chromosomes there are distinct peaks where Het (alive) > Het (dead). This suggests that genes along all three chromosomes have not been swept and that the pattern in Fig. 4 is not genome wide.

### Other associations not included in the *vgsc* cluster

In chromosome 1, no SNPs had −log_10_(prob) > 10. However, −log10(prob) with values of 8.9 to 9.9 occurred in protein FAM32A, synaptic vesicle glycoprotein 2B, GATOR complex protein NPRL2, neuropeptide F, transmembrane protease serine 9 and ceramide kinase (Fig. 2A). Nonsynonymous substitutions only occurred at the synaptic vesicle glycoprotein 2B, transcription factor and SPT20 aminopeptidase N (Table S5).

In chromosome 2, 54 SNPs with −log_10_(prob) > 10 were distributed among 44 genes. The highest −log_10_(prob) occurred at genes F‐box/WD repeat‐containing protein 7, G protein alpha subunit, zinc finger HIT domain‐containing protein, sialin and *S*‐acyltransferase (Fig. 2B). Nonsynonymous substitutions in this group [−log_10_(prob) > 10] were reported in six SNPs in genes coding for cholinesterase, actin‐binding protein IPP, zinc finger protein 836, Bardet–Biedl syndrome 2 protein, LOC5570539 and protein asteroid (Table S5).

In chromosome 3, high −log_10_(prob) SNPs that were swept by *vgsc* were scattered across genes coding for apolipophorin, chromatin‐remodelling complex ATPase, histone PARylation factor 1‐like, ubiquitin‐protein ligase E3C, histone H3‐like centromeric protein, proton‐coupled amino acid transporter CG1139, glutamyl aminopeptidase, trypsin‐2, LOC5573368 and CYP325T2 (Fig. 2C). A total of 29 nonsynonymous substitutions occurred in 22 genes, including *vgsc*, leucine‐rich repeat, proton‐coupled amino acid transporter‐like protein CG1139, large subunit GTPase, trypsin‐2, poly[ADP‐ribose] polymerase, GTPase, Era mitochondrial, glutamate receptor and apolipophorin (Table S5).

### Insecticide metabolism: detoxification genes

**Figure 5:**
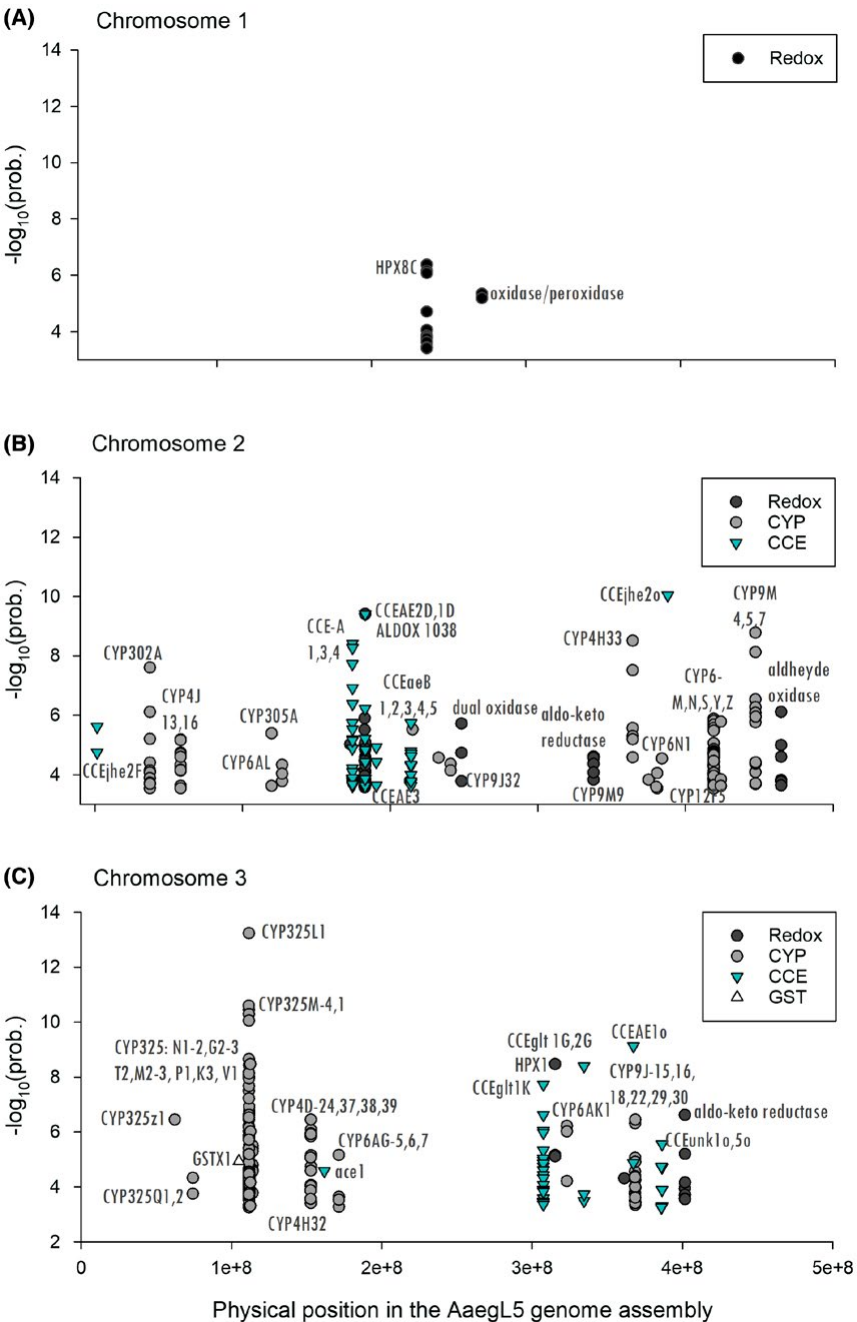
−log_10_(prob) of significant single‐nucleotide polymorphisms (SNPs) identified in detoxification genes. SNPs differing between knockdown‐resistant and susceptible *Aedes aegypti* are shown for chromosomes 1, 2 and 3 using the physical position of the AAeL5 physical map. We include synonymous and nonsynonymous substitutions, introns, 3′‐untranslated region (UTR), 5′‐UTR regions and noncoding RNAs. CYP, cytochrome P450 monooxygenases; CCE, carboxyl/choline esterases; GST, glutathione‐*S*‐transferase; Redox, reduction/oxidation enzymes.

We identified 268 insecticide detoxification genes (Strode *et al.*, 2008) in the AaeL5 assembly map and questioned which significant SNPs in our libraries were included in this categorical group. Genes with insecticide detoxification function surpassed the FDR cut‐off calculated for each chromosome. Fig. 5 shows the −log_10_(prob) calculated for the SNPs comprised in 2, 57 and 46 detoxification genes dispersed across chromosomes 1, 2 and 3 respectively. Overall, 62% of the SNPs are classify as CYP genes, 20% as CCE, 16% as Red/ox and 4% as GSTs. In chromosome 1, only 13 SNPs were significant, with 11 SNPs in HPX8C and two SNPs in an oxidase peroxidase (HPX6) (Fig. 5A). Most SNPs were annotated as synonymous substitutions, except for an S766A in HPX8C [−log_10_(prob) = 3.76]. Both genes are currently annotated as chorion peroxidases. Several peroxidase isozymes have been described in *Drosophila melanogaster* as cyclooxygenase‐like protein for both the actin filament bundle formation and the cortical actin strengthening during *Drosophila* oogenesis.

In chromosome 2, the highest associations were found in esterases CCEjhe2o, CCEae2D, CCEAE3A, 4A and 6A and cytochrome oxidases CYP9M7, CYP302A and CYP4H33 (Fig. 5B). Some of these genes were found as singletons (CYP302A, CYP4H33 and CCeJhe2o), whereas others were localized within clusters; for example, CYPJ13–16, CCEae1D–2D, CCEaeA‐1, 3, 4 and 6, CCEae‐B1, 2, 3, 4 and 5 and CYP9M‐4, 5–7 and CYP6‐M, N, S, Y and Z at the bottom of chromosome 2. Nonsynonymous substitutions occurred in *several* genes, two out of 18 SNPs were in CYP302A1, six nonsynonymous substitutions occurred in 27 SNPs at the CCEaeA‐1, 3, 4 and 6 cluster, and there were four out of 12 in the CCEaeB cluster and eight out of 12 in the CYP9M4 cluster (Table S5).

In chromosome 3, the highest association in this categorical group occurred at SNPs in genes CYP325T2, CYP325L1, CYP325G3 and CCEae10, with −log_10_(prob) ranging from 9 to 13.2. A total of 78% of the SNPs in this group (177 out of 228) consisted of cytochrome P450s. Interestingly, 109 SNPs were localized in a cluster of 14 CYP325 genes at the top of chromosome 3. These genes included CYP325L1, CYP325M‐1, 2, 3 and 4, CYP325G‐2 and 3, CYP325N1 and 2, CYP325P1, CYP325V1, CYP325T‐1 and 2 and CYP325K3. Downstream of this cluster (~30 Mbp), four CYP4D (24, 37, 38 and 39) and three CYP6AG (5, 6 and 7) were associated with deltamethrin resistance. At the q arm of chromosome 3, 24 SNPs were found only at esterase CCEglt1K, whereas three to four SNPs were identified in each of HPX1, CYP6AK1, CCEglt2G and CCEae10. A fourth cluster of significant CYP genes was comprised of 26 SNPs at CYP9–15, 16, 18, 22, 29 and 30, followed by two CCEunk10 and 5o, the latter annotated as cholinesterase.

Because previous studies focused mostly on gene expression, we compared our list of genes with significant SNPs with 22 studies that had measured gene expression in mosquitoes exposed to sublethal doses of insecticides (Poupardin *et al.*, 2008; 2010; 2012; Riaz *et al.*, 2009; David *et al.*, 2010). We also included published articles that compared insecticide‐resistant versus susceptible strains (Strode *et al.*, 2008; 2012; Marcombe *et al.*, 2009b; Saavedra‐Rodriguez *et al.*, 2012; 2014; David *et al.*, 2014; Reid *et al.*, 2014; Estep *et al.*, 2017) and, most recently, allelic variants (David *et al.*, 2014; Dusfour *et al.*, 2015; Faucon *et al.*, 2015; 2017). Fig. S3 shows the list of significant detoxification genes in our study and the expression that has been reported in previous studies. For example, in our study, CYP6N12 (LOC5571529) was significantly associated with deltamethrin resistance; this gene was significantly down/overexpressed in 12 studies, becoming a clear candidate for further study.

In analysing the proportion of polymorphic sites in detox genes versus all other genes, there was in fact a greater proportion of polymorphic sites in the detox genes. In chromosome 1, the proportion of polymorphic sites among detox genes was 0.078 (306/3932), compared with 0.052 (9369/180 563) in all other genes, and this difference was significant with Fisher's exact test (*P* = 6.906 × 10^−13^). In chromosome 2, the proportion of polymorphic sites among detox genes was 0.053 (7126/134 908), compared with 0.048 (221 876/4 657 182) in all other genes (*P* = 1 × 10^−16^). In chromosome 3, the proportion of polymorphic sites among detox genes was 0.055 (3846/69 684), compared with 0.046 (109 310/2 360 622) (*P* = 1 × 10^−16^).

Furthermore, Campbell *et al.* (2019) analysed a compilation of high‐throughput sequencing data from three pyrethroid treatment groups and interrogated them to identify functional categories that were overrepresented among all coding genes. Of eight functional categories, three were overrepresented, as calculated by a hypergeometric test, which tests the probability of a gene being represented more often than expected by chance. The three overrepresented groups were metabolism, transport and signalling. The detox gene subset would encompass both the metabolic category and the redox category, which was not overrepresented.

### Genes potentially involved in behaviour and reduced penetration

Recently, a neuro‐transcriptome of 576 genes related to the mosquito nervous system and involved in chemosensory behaviour was described (Matthews *et al.*, 2016). The list includes neuropeptides, neuropeptide receptors, odorant receptors, odorant binding proteins, gustatory receptors, ionotropic receptors, pickpocket proteins and others. In our study, 625 significant SNPs included in 149 genes matched to this list, mostly represented by odorant receptors (170 SNPs), ionotropic (Matthews *et al.*, 2016) receptors (177 SNPs), pickpocket proteins (97 SNPs) and gustatory receptors (85 SNPs). Also, acetylcholinesterase receptors (20 SNPs), GABA receptors (eight SNPs) and neuropeptide receptors (23) were identified. The highest associations in chromosome 2 occurred in gustatory receptor 18, general odorant‐binding protein 67, odorant receptor 69 and acetylcholine receptor, with −log_10_(prob) ranging between 6.75 to 8.53. In chromosome 3, the largest association occurred at ionotropic receptor 41d, 93a and general odorant‐binding protein 72, with −log_10_(prob) ranging between 9.3 to 11.05.

Around 260 genes in the AaeL5 have descriptions as cuticle, endocuticle, chitin, chitinase and melanization. In our analysis, 125 SNPs belong to genes within these categories. In chromosome 1, a single melanization protease had six significant SNPs, with −log_10_(prob) ranging between 3.9 and 6.9. In chromosome 2, cuticle protein 8, pupal cuticle protein 36, probable chitinase 10, adult‐specific cuticular protein ACP‐20 had −log_10_(prob) ranging from 7.7 to 10.5. In chromosome 3, a probable chitinase, cuticle protein 16.5 and flexible cuticle protein 12‐like had −log_10_(prob) of 5.6, 5.9 and 9.4 respectively.

## Discussion

With increased insecticide spraying to protect public health, the detection and characterization of resistance loci are a crucial requisite for adequate monitoring of the rise of insecticide resistance that could hamper vector control efforts. Currently, kdr is a major mechanism of pyrethroid resistance; around 11 amino acid replacements in *vgsc* have been associated with this trait in *A. aegypti*, and quantification of their frequencies in field populations is becoming a common practice to monitor pyrethroid resistance. Unfortunately, continued use of pyrethroids in the field is leading to the fixation of kdr mutations and possibly the selection of novel or compensatory mutations in *vgsc* and other genes associated with pyrethroid resistance. Identifying these novel or compensatory mutations is necessary to comprehensively monitor pyrethroid resistance in field populations. In this study, 52–80% of *vgsc* was sequenced across libraries (out of 7794 nucleotides covered by enrichment probes covering exons 5–30 and introns 4–29) and 19 SNPs were significantly associated with deltamethrin resistance. Notably, three nonsynonymous mutations in *vgsc* had significant association. Two are novel mutations, and their role in pyrethroid resistance is unknown. Contrastingly, V410L was highly associated with resistance in our study, bringing further evidence in its role in pyrethroid resistance (Haddi *et al.*, 2017). We recently tracked V410L in a population from Mexico collected in 2002, and frequencies have increased in the last decade in strong linkage disequilibrium to mutations I1016 and C1534 (Saavedra‐Rodriguez *et al.*, 2018). Interestingly, I1016 and C1534 were *not* significantly associated with deltamethrin resistance in our study. To understand this observation, we individually genotyped mosquitoes included in our libraries and calculated allele frequencies of I1016 and C1534. I1016 had identical frequencies to those found in L410; however, coverage (>25 reads) was not reached at this site. The improvement of library preparation and DNA pool optimization will address the identification of this locus in association with pyrethroid resistance. Contrastingly, the frequency of C1534 was high (>0.9) in both susceptible and resistant libraries, so that −log_10_(prob) values were not high enough to surpass the chromosome FDR threshold. In Mexico, strong selection has pushed C1534 to fixation in many field populations (Vera‐Maloof *et al*., 2014). Therefore, the use of C1534 as a marker for pyrethroid resistance is no longer informative in Mexico, even though the role of C1534 in pyrethroid resistance has been validated by electrophysiology studies (Du *et al.*, 2013; Haddi *et al.*, 2017). Furthermore, C1534 appears to be necessary for other mutations (I1016) to occur and increase in frequency in the population (Vera‐Maloof *et al*., 2015).

Interestingly, we identified a possible genetic sweep driven by selection of *vgsc* nonsynonymous mutations associated with pyrethroid resistance. Similar genetic sweeps have been reported in *D. melanogaster* driven by selection of acetylcholinesterase mutations associated with carbamate and organophosphate resistance and by DDT resistance mediated by an insertion of a transposon in the 5′ regulatory region of CYP6g1 (Garud *et al.*, 2015). Similarly, strong signals of recent positive selection at several genes that are known to have a role in resistance have been identified in *Anopheles*, including *vgsc*, a cluster of GST genes including *Gste2* (implicated in the metabolism of DDT and pyrethroids), and *Cyp6p*, a cluster of genes that include *Cyp6p3*, which is upregulated in permethrin and bendiocarb‐resistant mosquitoes (Anopheles gambiae 2017 Genomes Consortium, 2017). In *A. aegypti*, evidence of a genetic sweep (or hitchhiking effect) was demonstrated in a region of chromosome 1 that contains organophosphate‐resistance‐conferring genes (Yan *et al.*, 1998). The genetic sweep was reflected by low DNA polymorphisms and gene deletions for loci surrounding the EST‐4 locus gene region. In contrast, a hitchhiking effect associated with DDT was not evident for the para (*vgsc*) locus, in which heterozygosity was similar to other regions in the genome. This result was consistent with the hypothesis that, in the years since DDT was abandoned, the populations have had time to re‐equilibrate. Luckily, with the *A. aegypti* physical map completed, we can test the gain/loss of genomic variability in populations exposed to different pressures of insecticides.

The role of metabolism in conferring pyrethroid resistance is unquestionable. Even if kdr mutations at *vgsc* protect mosquitoes from knockdown, pyrethroids are metabolized before reaching *vgsc* or after dissociation from *vgsc* by different enzymes to less toxic and more hydrophilic metabolites for excretion. The metabolism of pyrethroids occurs by ester hydrolysis (by CCE) and oxidation at methyl, methylene, alkenyl or aryl substituents (CYP), and ≥80 metabolites have been identified from *cis* and *trans*‐permethrin (Casida *et al.*, 1983). To date, fewer than 10 genes have been functionally characterized and confirmed to metabolize pyrethroids in *A. aegypti*, including members of the CYP9 family (Stevenson *et al.*, 2012), CYP9M6 and CYP6BB2 (Kasai *et al.*, 2014), whereas other enzymes, such as CYP6Z8 and the Red/ox (aldehyde dehydrogenases), catalyse the oxidation of permethrin intermediates (Chandor‐Proust *et al.*, 2013; Lumjuan *et al.*, 2014). Noticeably, genes CYP6Z8, CYP9J26–28 and CYP9M6 belong to clusters of tandem genes. In our study, a large cluster of CYP325 genes at the p arm of chromosome 3 was strongly associated with deltamethrin. Although, the physiological function of the CYP325 in insects still remains unclear, some CYP325s (eg CYP325‐E, K and G) are conserved among mosquito species, some only expanded in the *Culex* and *Aedes* genomes (CYP325X and Y) and some are species specific (Yan *et al.*, 2012). In our review, at least 13 CYP325 genes (Fig. S3) have been associated with changes of expression (two to threefold) in insecticide‐resistant *A. aegypti*. In one study, CYP325N1 was 313‐fold more expressed in a pyrethroid‐resistant strain from Puerto Rico (Estep *et al.*, 2017). In other insects, only CYP325A3 was reported in a permethrin‐resistant strain of *Anopheles gambiae* (David *et al.*, 2005); however, no clear orthologues were found in *Aedes* or *Drosophila*. Further research of the CYP325 cluster might help elucidate models for mechanisms of cluster expression and adaptation to specific insecticides, if any.

Similarly, identification and isolation of specific esterases that metabolize pyrethroids is ongoing, mainly because esterases have unspecific substrates that complicate protein isolation. In this study, several clustered CCE genes in chromosomes 2 (CCEaeA, CCEaeB and CCEaeD) and 3 (CCEunk and CCEglt) showed high association with deltamethrin resistance. CCEaeA family members are commonly reported in expression studies (Fig. S3). Genes from this cluster showed strong amplification in two deltamethrin‐resistant strains from Thailand but were not present in two pyrethroid‐resistant strains from South America (Faucon *et al.*, 2015). Similarly, CCEae3A and 6A have been associated with temephos resistance in an *A. aegypti* strain for Thailand that was also resistant to pyrethroids (Poupardin *et al.*, 2014). Although amplification of these CCE clusters are a possible mechanism to overexpress enzymes that metabolize insecticides, our study and others (Faucon *et al.*, 2015; 2017) have also identified allele variants within these genes. The roles of these nonsynonymous mutations in catalytic activity is of great interest. For example, amino acid substitutions increased diazinon hydrolysis by esterase E7 in *Lucilia cuprina* (Newcomb *et al.*, 1997) and *M. domestica* (Claudianos *et al.*, 1999), whereas a different substitution in the same gene was responsible for malathion resistance in *L. cuprina* (Campbell *et al.*, 1998). Characterization of CCE genes and their allelic variants will increase our knowledge of possible routes of metabolism.

We have mapped genomic regions associated with deltamethrin resistance in mosquitoes from Mexico. Our results validate the major role of *vgsc* in pyrethroid resistance, but fixation of replacement mutations in *vgsc* suggest that kdr is becoming well established in field populations. Additional mechanisms of deltamethrin resistance might play increasingly important roles in the future, following genomic changes in field populations that are routinely pressured with insecticides. It is therefore necessary to identify genes and possible mechanisms being selected by pyrethroids in general. So far, we have identified several detoxification genes that are highly variable in resistant mosquitoes. Research to further associate allelic variants with deltamethrin resistance is ongoing.

## Experimental procedures

### Mosquito samples, bioassays and DNA isolation

The *A. aegypti* population Viva Caucel from Yucatán State in southern Mexico (longitude −89.71827, latitude 20.99827) was collected in 2011 by Universidad Autónoma de Yucatán. This mosquito population was under field selection by a type‐1 pyrethroid (permethrin) in vector control programmes from 1999 to 2010. We calculated the deltamethrin concentration to kill 50% of the mosquitoes (LC_50_) by bottle bioassays to establish the level of resistance in the Viva Caucel population (F_3_) relative to the susceptible New Orleans (NO). Around 20 non‐blood‐fed females (2–3 days old) of the Viva Caucel F_3_ and NO were exposed to seven deltamethrin (Chem Service, West Chester, PA, USA) concentrations for 1 h, in three replicates. Mortality was scored following a recovery time of 24 h. The deltamethrin LC_50_ at 24 h was 47.6‐fold higher in the Viva Caucel population than in the NO susceptible strain (10.49 µg vs. 0.22 µg respectively) (Fig. S2). The NO strain was not used further in this study.

To discriminate the deltamethrin‐resistant and susceptible individuals within the Viva Caucel population, 390 adult mosquitoes were exposed to 3 µg of deltamethrin for 1 h. This concentration is below the LC_50_ calculated at 24 h post‐exposure; however, 3 µg allowed us to discriminate the three phenotypes at 4 h of observation (instead of 24 h) while ensuring good DNA quality in ‘susceptible’ mosquitoes. Additionally, 4 h is a reasonable time for recovery, since longer periods increase the chances of predation and dehydration in knocked‐down mosquitoes in the field.

The bioassay was performed by aspirating groups of 50 non‐blood‐fed female mosquitoes (3–4 days old) into deltamethrin‐coated bottles for 1 h. After this time, active mosquitoes were transferred to 1 pint cardboard cups and placed into an incubator (28 °C and 70% humidity) for 4 h. These mosquitoes constituted the ‘knockdown‐resistant’ group. Knocked‐down mosquitoes were then transferred to a second cardboard cup. Four hours later, newly recovered mosquitoes were aspirated, frozen and labelled as ‘recovered’. These were not used in the current study. The knocked‐down mosquitoes that remained inactive at 4 h post‐treatment were scored as ‘susceptible’. DNA was isolated from individual mosquitoes that were categorized as either resistant or susceptible (Black and DuTeau, 1997) and resuspended in 150 µl of TE buffer (10 mM Tris hydrochloride, 1 mM ethylenediaminetetraacetic acid pH 8.0).

### Sample pooling and library preparation

We constructed four genomic DNA libraries. A library was generated from the DNA of 25 knockdown‐resistant females (Fig. 1), and this was replicated in a second set of 25 mosquitoes. The same was done with susceptible females (susceptible replicates 1 and 2) (Fig. 1). Before pooling, DNA from each individual mosquito was quantified using the Quant‐IT Pico Green kit (Invitrogen, Eugene, OR, USA), and ~40 ng from each individual DNA sample was used for a final DNA pool of 1 µg. Pooled DNA was sheared and fragmented by sonication to obtain fragments between 300 and 500 bp (Covaris Ltd, Brighton, UK). We prepared one library for each of the four DNA pools following the Low Sample (LS) protocol from the Illumina TrueSeqDNA PCR‐Free Sample preparation guide (Illumina, San Diego, CA, USA).

### Whole exome hybrid capture

Because of the large 1.38 Gbp size of the *A. aegypti* genome (Nene *et al.*, 2007), we used an exome capture method. To identify the proportion of L5 genes covered by the capture probes, the analogous L5 probe sequences were blasted (blastn) against the L5 reference transcriptome. The returned genes were cross‐checked against the total number of L5 genes (18 081). Thus, 77% (13 941/18 081) of L5 genes were accounted for among the probe set. We performed an exome‐capture hybridization to enrich for coding sequences using custom SeqCap EZ Developer probes (Roche NimbleGen Inc., Madison, WI, USA). Probes covered protein coding sequences (not including UTRs) in the AaegL1.3 genebuild using the exonic coordinates specified previously (Juneja *et al.*, 2015). In total, 26.7 Mb of the genome (2%) containing 15 735 genes was targeted for enrichment. TruSeq libraries were hybridized to the probes using the xGen®Lock®Down recommendations (Integrated DNA Technologies, Coralville, Iowa, USA). The targeted DNA was eluted and amplified (10‐15 cycles) before being sequenced on one flow cell of a 100 bp HiSeq Rapid‐duo paired‐end sequencing run (Illumina) performed by the Centers for Disease Control and Prevention (Atlanta, GA, USA).

### Analysis pipeline

The raw sequence files (*.fastq) for each pair‐ended gDNA library were aligned to a custom reference physical map generated from the assembly AaegL5 (Matthews *et al.*, 2018) using the package gsnap (Genomic Short‐read Nucleotide Alignment; Wu and Nacu, 2010), which performed an SNP‐tolerant alignment of the resulting (39–42) × 10^6^ paired reads/library. Additional processing details for our in‐house fortran pipeline are provided in Dickson *et al.* (2017). Briefly, we removed SNPs that were significantly different between replicates. Next, we grouped replicates that share the same sites together to build contingency tables and the heterogeneity *χ* ^2^ was calculated with (*n* − 1) nucleotides degrees of freedom. The probability derived from that analysis was −log_10_ transformed to provide a −log_10_(prob) value. A Benjamini–Hochberg correction for FDR (Benjamini and Hochberg, 1995) was applied for chromosomes 1, 2 and 3 separately (*α* = 0.01) using sas.

### Validation of nonsynonymous substitution V410L in *vgsc*

We determined the individual genotype of the mosquitoes used in our four libraries (*n* = 100) at two nonsynonymous substitutions in the *vgsc*. We performed melting curve analyses of allele‐specific PCR products to genotype at locus V1016I (Saavedra‐Rodriguez *et al.*, 2007) and a recently reported mutation at locus V410L (Haddi *et al.*, 2017) following an allele‐specific PCR described in Saavedra‐Rodriguez *et al.* (2018).

## Data availability

All fastq files are available at the National Center for Biotechnology Information Sequence Read Archive (https://www.ncbi.nlm.nih.gov/sra), Bioproject accession no. PRJNA393171. VCDA deltamethrin‐resistant replicates 1 and 2 are SRR5805467 and SRR5805466 respectively. VCDD deltamethrin‐susceptible replicates 1 and 2 are SRR5805469 and SRR5805468 respectively.

## Supporting information


**Figure S1.** Distribution of the differences in heterozygosity in knockdown resistant versus susceptible mosquitoes (H_exp_ alive (HETA)   H_exp_ dead (HETD)) at each significant SNP in chromosomes 1, 2 and 3.

**Figure S2.** Deltamethrin dose response curve for female *Ae. aegypti* from Viva Caucel and the reference strain from New Orleans. Exposure was performed using bottles coated with increasing concentrations of deltamethrin. Exposure time was 1 h and mortality was recorded at 24 h.

**Figure S3.** List of significant detoxification genes in our study and the expression that has been reported in previous studies. Upregulated genes are highlighted in orange, downregulated are highlighted in blue, downregulated or upregulated in the same study are highlighted in grey. A number represents the number of polymorphisms found at a particular SNP in that gene.

**Table S1.** Number of reads aligned AaeL5 across chromosome 1, 2 and 3 for each replicated libraries. The number of common reads between replicates and among groups (knockdown resistant vs susceptible) is shown.

**Table S2.** List of Individual SNP, physical position of each SNP, gene description, mutation, frequencies of alternant nucleotide in resistant and susceptible groups,  log_10_(prob), nucleotide, type of SNP, substitution, amino acid residue and codon position of each SNP for chromosome 1.

**Table S3.** List of Individual SNP, physical position of each SNP, gene description, mutation, frequencies of alternant nucleotide in resistant and susceptible groups,  log_10_(prob), nucleotide, type of SNP, substitution, amino acid residue and codon position of each SNP for chromosome 2.

**Table S4.** List of Individual SNP, physical position of each SNP, gene description, mutation, frequencies of alternant nucleotide in resistant and susceptible groups,  log_10_(prob), nucleotide, type of SNP, substitution, amino acid residue and codon position of each SNP for chromosome 3.

**Table S5.** List of non synonymous mutations in deltamethrin resistant *Ae. aegypti*. The genes, description, SNPID, residue number, frequencies in resistant and susceptible and  log_10_(prob) is shown for chromosomes 1, 2 and 3.

## References

[imb12575-bib-0001] Anopheles Gambiae Genomes Consortium . (2017) Genetic diversity of the African malaria vector *Anopheles gambiae* . Nature, 552, 96–100. 29186111 10.1038/nature24995 PMC6026373

[imb12575-bib-0002] Aponte, H.A. , Penilla, R.P. , Dzul‐Manzanilla, F. , Che‐Mendoza, A. , López, A.D. , Solis, F. et al. (2013) The pyrethroid resistance status and mechanisms in *Aedes aegypti* from the Guerrero State, Mexico. Pesticide Biochemistry and Physiology, 107, 226–234.

[imb12575-bib-0003] Balabanidou, V. , Grigoraki, L. and Vontas, J. (2018) Insect cuticle: a critical determinant of insecticide resistance. Current Opinion in Insect Science, 27, 68–74. 30025637 10.1016/j.cois.2018.03.001

[imb12575-bib-0004] Bariami, V. , Jones, C.M. , Poupardin, R. , Vontas, J. and Ranson, H. (2012) Gene amplification, ABC transporters and cytochrome P450s: unraveling the molecular basis of pyrethroid resistance in the dengue vector, *Aedes aegypti* . PLoS Neglected Tropical Diseases, 6, e1692. 22720108 10.1371/journal.pntd.0001692 PMC3373657

[imb12575-bib-0005] Benjamini, Y. and Hochberg, Y. (1995) Controlling the false discovery rate – a practical and powerful approach to multiple testing. Journal of the Royal Statistical Society Series B – Methodological, 57, 289–300.

[imb12575-bib-0006] Bingham, G. , Strode, C. , Tran, L. , Khoa, P.T. and Jamet, H.P. (2011) Can piperonyl butoxide enhance the efficacy of pyrethroids against pyrethroid‐resistant *Aedes aegypti*? Tropical Medicine & International Health, 16, 492–500. 21324051 10.1111/j.1365-3156.2010.02717.x

[imb12575-bib-0007] Black IV, W.C. and Duteau, N.M. (1997) RAPD‐PCR and SSCP analysis for insect population genetic studies In: Crampton J.M., Beard C.B. and Louis C. (Eds) The Molecular Biology of Insect Disease Vectors: A Methods Manual. New York, NY: Chapman and Hall, pp. 361–373.

[imb12575-bib-0008] Campbell, C.L. , Saavedra‐Rodriguez, K. , Kubik, T.D. , Lenhart, A. , Lozano‐Fuentes, S. and Black, W.C. (2019) Vgsc‐interacting proteins are genetically associated with pyrethroid resistance in *Aedes aegypti* . PLoS ONE, 14(1), e0211497. 30695054 10.1371/journal.pone.0211497 PMC6350986

[imb12575-bib-0009] Campbell, P.M. , Newcomb, R.D. , Russell, R.J. and Oakeshott, J.G. (1998) Two different amino acid substitutions in the ali‐esterase, E3, confer alternative types of organophosphorus insecticide resistance in the sheep blowfly, *Lucilia cuprina* . Insect Biochemistry and Molecular Biology, 28, 139–150.

[imb12575-bib-0010] Casida, J.E. , Gammon, D.W. , Glickman, A.H. and Lawrence, L.J. (1983) Mechanisms of selective action of pyrethroid insecticides. Annual Review of Pharmacology and Toxicology, 23, 413–438. 10.1146/annurev.pa.23.040183.002213 6347050

[imb12575-bib-0011] Chandor‐Proust, A. , Bibby, J. , Regent‐Kloeckner, M. , Roux, J. , Guittard‐Crilat, E. , Poupardin, R. et al. (2013) The central role of mosquito cytochrome P450 CYP6Zs in insecticide detoxification revealed by functional expression and structural modelling. The Biochemical Journal, 455, 75–85. 23844938 10.1042/BJ20130577 PMC3778711

[imb12575-bib-0012] Claudianos, C. , Russell, R.J. and Oakeshott, J.G. (1999) The same amino acid substitution in orthologous esterases confers organophosphate resistance on the house fly and a blowfly. Insect Biochemistry and Molecular Biology, 29, 675–686. 10451921 10.1016/s0965-1748(99)00035-1

[imb12575-bib-0013] Da‐Cunha, M.P. , Lima, J.B. , Brogdon, W.G. , Moya, G.E. and Valle, D. (2005) Monitoring of resistance to the pyrethroid cypermethrin in Brazilian *Aedes aegypti* (Diptera: Culicidae) populations collected between 2001 and 2003. Memorias do Instituto Oswaldo Cruz, 100, 441–444. 16113895 10.1590/s0074-02762005000400017

[imb12575-bib-0014] David, J.P. , Coissac, E. , Melodelima, C. , Poupardin, R. , Riaz, M.A. , Chandor‐Proust, A. et al. (2010) Transcriptome response to pollutants and insecticides in the dengue vector *Aedes aegypti* using next‐generation sequencing technology. BMC Genomics, 11, 216. 20356352 10.1186/1471-2164-11-216 PMC2867825

[imb12575-bib-0015] David, J.P. , Faucon, F. , Chandor‐Proust, A. , Poupardin, R. , Riaz, M.A. , Bonin, A. et al. (2014) Comparative analysis of response to selection with three insecticides in the dengue mosquito *Aedes aegypti* using mRNA sequencing. BMC Genomics, 15, 174. 24593293 10.1186/1471-2164-15-174 PMC4029067

[imb12575-bib-0016] David, J.P. , Strode, C. , Vontas, J. , Nikou, D. , Vaughan, A. , Pignatelli, P.M. et al. (2005) The *Anopheles gambiae* detoxification chip: a highly specific microarray to study metabolic‐based insecticide resistance in malaria vectors. Proceedings of the National Academy of Sciences of the United States of America, 102, 4080–4084. 15753317 10.1073/pnas.0409348102 PMC554807

[imb12575-bib-0017] Dickson, L.B. , Campbell, C.L. , Juneja, P. , Jiggins, F.M. , Sylla, M. and Black, W.C.T. (2017) Exon‐enriched libraries reveal large genic differences between *Aedes aegypti* from Senegal, West Africa, and populations outside Africa. G3 (Bethesda), 7, 571–582. 28007834 10.1534/g3.116.036053 PMC5295602

[imb12575-bib-0018] Du, Y. , Nomura, Y. , Satar, G. , Hu, Z. , Nauen, R. , He, S.Y. et al. (2013) Molecular evidence for dual pyrethroid‐receptor sites on a mosquito sodium channel. Proceedings of the National Academy of Sciences of the United States of America, 110, 11785–11790. 23821746 10.1073/pnas.1305118110 PMC3718148

[imb12575-bib-0019] Dusfour, I. , Zorrilla, P. , Guidez, A. , Issaly, J. , Girod, R. , Guillaumot, L. et al. (2015) Deltamethrin resistance mechanisms in *Aedes aegypti* populations from three French overseas territories worldwide. PLoS Neglected Tropical Diseases, 9, e0004226. 26588076 10.1371/journal.pntd.0004226 PMC4654492

[imb12575-bib-0020] Estep, A.S. , Sanscrainte, N.D. , Waits, C.M. , Louton, J.E. and Becnel, J.J. (2017) Resistance status and resistance mechanisms in a strain of *Aedes aegypti* (Diptera: Culicidae) from Puerto Rico. Journal of Medical Entomology, 54, 1643–1648. 28981681 10.1093/jme/tjx143

[imb12575-bib-0021] Faucon, F. , Dusfour, I. , Gaude, T. , Navratil, V. , Boyer, F. , Chandre, F. et al. (2015) Identifying genomic changes associated with insecticide resistance in the dengue mosquito *Aedes aegypti* by deep targeted sequencing. Genome Research, 25, 1347–1359. 26206155 10.1101/gr.189225.115 PMC4561493

[imb12575-bib-0022] Faucon, F. , Gaude, T. , Dusfour, I. , Navratil, V. , Corbel, V. , Juntarajumnong, W. et al. (2017) In the hunt for genomic markers of metabolic resistance to pyrethroids in the mosquito *Aedes aegypti*: an integrated next‐generation sequencing approach. PLoS Neglected Tropical Diseases, 11, e0005526. 28379969 10.1371/journal.pntd.0005526 PMC5393893

[imb12575-bib-0023] Flores, A.E. , Ponce, G. , Silva, B.G. , Gutierrez, S.M. , Bobadilla, C. , Lopez, B. et al. (2013) Wide spread cross resistance to pyrethroids in *Aedes aegypti* (Diptera: Culicidae) from Veracruz State Mexico. Journal of Economic Entomology, 106, 959–969. 23786088 10.1603/ec12284 PMC3980443

[imb12575-bib-0024] Garud, N.R. , Messer, P.W. , Buzbas, E.O. and Petrov, D.A. (2015) Recent selective sweeps in North American *Drosophila melanogaster* show signatures of soft sweeps. PLoS Genetics, 11, e1005004. 25706129 10.1371/journal.pgen.1005004 PMC4338236

[imb12575-bib-0025] Grisales, N. , Poupardin, R. , Gomez, S. , Fonseca‐Gonzalez, I. , Ranson, H. and Lenhart, A. (2013) Temephos resistance in *Aedes aegypti* in Colombia compromises dengue vector control. PLoS Neglected Tropical Diseases, 7, e2438. 24069492 10.1371/journal.pntd.0002438 PMC3777894

[imb12575-bib-0026] Haddi, K. , Tome, H.V.V. , Du, Y. , Valbon, W.R. , Nomura, Y. , Martins, G.F. et al. (2017) Detection of a new pyrethroid resistance mutation (V410L) in the sodium channel of *Aedes aegypti*: a potential challenge for mosquito control. Scientific Reports, 7, 46549. 28422157 10.1038/srep46549 PMC5396194

[imb12575-bib-0027] Harris, A.F. , Rajatileka, S. and Ranson, H. (2010) Pyrethroid resistance in *Aedes aegypti* from Grand Cayman. American Journal of Tropical Medicine and Hygiene, 83, 277–284. 20682868 10.4269/ajtmh.2010.09-0623 PMC2911171

[imb12575-bib-0028] Hemingway, J. and Karunaratne, S.H. (1998) Mosquito carboxylesterases: a review of the molecular biology and biochemistry of a major insecticide resistance mechanism. Medical and Veterinary Entomology, 12, 1–12. 9513933 10.1046/j.1365-2915.1998.00082.x

[imb12575-bib-0029] Hirata, K. , Komagata, O. , Itokawa, K. , Yamamoto, A. , Tomita, T. and Kasai, S. (2014) A single crossing‐over event in voltage‐sensitive Na^+^ channel genes may cause critical failure of dengue mosquito control by insecticides. PLoS Neglected Tropical Diseases, 8, e3085. 25166902 10.1371/journal.pntd.0003085 PMC4148226

[imb12575-bib-0030] Juneja, P. , Ariani, C.V. , Ho, Y.S. , Akorli, J. , Palmer, W.J. , Pain, A. et al. (2015) Exome and transcriptome sequencing of *Aedes aegypti* identifies a locus that confers resistance to *Brugia malayi* and alters the immune response. PLoS Pathogens, 11, e1004765. 25815506 10.1371/journal.ppat.1004765 PMC4376896

[imb12575-bib-0031] Kasai, S. , Komagata, O. , Itokawa, K. , Shono, T. , Ng, L.C. , Kobayashi, M. et al. (2014) Mechanisms of pyrethroid resistance in the dengue mosquito vector, *Aedes aegypti*: target site insensitivity, penetration, and metabolism. PLoS Neglected Tropical Diseases, 8, e2948. 24945250 10.1371/journal.pntd.0002948 PMC4063723

[imb12575-bib-0032] Lumjuan, N. , Wicheer, J. , Leelapat, P. , Choochote, W. and Somboon, P. (2014) Identification and characterisation of *Aedes aegypti* aldehyde dehydrogenases involved in pyrethroid metabolism. PLoS ONE, 9, e102746. 25047125 10.1371/journal.pone.0102746 PMC4105619

[imb12575-bib-0033] Marcombe, S. , Carron, A. , Darriet, F. , Etienne, M. , Agnew, P. , Tolosa, M. et al. (2009a) Reduced efficacy of pyrethroid space sprays for dengue control in an area of Martinique with pyrethroid resistance. American Journal of Tropical Medicine and Hygiene, 80, 745–751. 19407118

[imb12575-bib-0034] Marcombe, S. , Poupardin, R. , Darriet, F. , Reynaud, S. , Bonnet, J. , Strode, C. et al. (2009b) Exploring the molecular basis of insecticide resistance in the dengue vector *Aedes aegypti*: a case study in Martinique Island (French West Indies). BMC Genomics, 10, 494. 19857255 10.1186/1471-2164-10-494 PMC2770535

[imb12575-bib-0035] Marcombe, S. , Mathieu, R.B. , Pocquet, N. , Riaz, M.A. , Poupardin, R. , Selior, S. et al. (2012) Insecticide resistance in the dengue vector *Aedes aegypti* from Martinique: distribution, mechanisms and relations with environmental factors. PLoS ONE, 7, e30989. 22363529 10.1371/journal.pone.0030989 PMC3283601

[imb12575-bib-0036] Matthews, B.J. , Dudchenko, O. , Kingan, S. , Koren, S. , Antoshechkin, I. , Crawford, J.E. et al. (2018). Improved *Aedes aegypti* mosquito reference genome assembly enables biological discovery and vector control. Nature, (in press). 563, 501–507. 30429615 10.1038/s41586-018-0692-z PMC6421076

[imb12575-bib-0037] Matthews, B.J. , McBride, C.S. , Degennaro, M. , Despo, O. and Vosshall, L.B. (2016) The neurotranscriptome of the *Aedes aegypti* mosquito. BMC Genomics, 17, 32. 26738925 10.1186/s12864-015-2239-0 PMC4704297

[imb12575-bib-0038] Nene, V. , Wortman, J.R. , Lawson, D. , Haas, B. , Kodira, C. , Tu, Z.J. et al. (2007) Genome sequence of *Aedes aegypti*, a major arbovirus vector. Science, 316, 1718–1723. 17510324 10.1126/science.1138878 PMC2868357

[imb12575-bib-0039] Newcomb, R.D. , Campbell, P.M. , Ollis, D.L. , Cheah, E. , Russell, R.J. and Oakeshott, J.G. (1997) A single amino acid substitution converts a carboxylesterase to an organophosphorus hydrolase and confers insecticide resistance on a blowfly. Proceedings of the National Academy of Sciences of the United States of America, 94, 7464–7468. 9207114 10.1073/pnas.94.14.7464 PMC23844

[imb12575-bib-0040] Poupardin, R. , Reynaud, S. , Strode, C. , Ranson, H. , Vontas, J. and David, J.P. (2008) Cross‐induction of detoxification genes by environmental xenobiotics and insecticides in the mosquito *Aedes aegyp*ti: impact on larval tolerance to chemical insecticides. Insect Biochemistry and Molecular Biology, 38, 540–551. 18405832 10.1016/j.ibmb.2008.01.004

[imb12575-bib-0041] Poupardin, R. , Riaz, M.A. , Vontas, J. , David, J.P. and Reynaud, S. (2010) Transcription profiling of eleven cytochrome P450s potentially involved in xenobiotic metabolism in the mosquito *Aedes aegypti* . Insect Molecular Biology, 19, 185–193. 20041961 10.1111/j.1365-2583.2009.00967.x

[imb12575-bib-0042] Poupardin, R. , Riaz, M.A. , Jones, C.M. , Chandor‐Proust, A. , Reynaud, S. and David, J.P. (2012) Do pollutants affect insecticide‐driven gene selection in mosquitoes? Experimental evidence from transcriptomics. Aquatic Toxicology, 114–115, 49–57. 10.1016/j.aquatox.2012.02.001 22406618

[imb12575-bib-0043] Poupardin, R. , Srisukontarat, W. , Yunta, C. and Ranson, H. (2014) Identification of carboxylesterase genes implicated in temephos resistance in the dengue vector *Aedes aegypti* . PLoS Neglected Tropical Diseases, 8, e2743. 24651719 10.1371/journal.pntd.0002743 PMC3961196

[imb12575-bib-0044] Reid, W.R. , Thornton, A. , Pridgeon, J.W. , Becnel, J.J. , Tang, F. , Estep, A. et al. (2014) Transcriptional analysis of four family 4 P450s in a Puerto Rico strain of *Aedes aegypti* (Diptera: Culicidae) compared with an Orlando strain and their possible functional roles in permethrin resistance. Journal of Medical Entomology, 51, 605–615. 24897853 10.1603/me13228

[imb12575-bib-0045] Riaz, M.A. , Poupardin, R. , Reynaud, S. , Strode, C. , Ranson, H. and David, J.P. (2009) Impact of glyphosate and benzo[*a*]pyrene on the tolerance of mosquito larvae to chemical insecticides. Role of detoxification genes in response to xenobiotics. Aquatic Toxicology, 93, 61–69. 19419775 10.1016/j.aquatox.2009.03.005

[imb12575-bib-0046] Rinkevich, F.D. , Du, Y. and Dong, K. (2013) Diversity and convergence of sodium channel mutations involved in resistance to pyrethroids. Pesticide Biochemistry and Physiology, 106, 93–100. 24019556 10.1016/j.pestbp.2013.02.007 PMC3765034

[imb12575-bib-0047] Saavedra‐Rodriguez, K. , Vera Maloof, F. , Campbell, C.L. , Garcia‐Rejon, J. , Lenhart, A. , Penilla, P. et al. (2018) Parallel evolution of *vgsc* mutations at domains IS6, IIS6 and IIIS6 in pyrethroid resistant *Aedes aegypti* from Mexico. Scientific Reports, 8, 6747. 29712956 10.1038/s41598-018-25222-0 PMC5928250

[imb12575-bib-0048] Saavedra‐Rodriguez, K. , Strode, C. , Flores, A.E. , Garcia‐Luna, S. , Reyes‐Solis, G. , Ranson, H. et al. (2014) Differential transcription profiles in *Aedes aegypti* detoxification genes after temephos selection. Insect Molecular Biology, 23, 199–215. 24299217 10.1111/imb.12073 PMC4091897

[imb12575-bib-0049] Saavedra‐Rodriguez, K. , Suarez, A.F. , Salas, I.F. , Strode, C. , Ranson, H. , Hemingway, J. et al. (2012) Transcription of detoxification genes after permethrin selection in the mosquito *Aedes aegypti* . Insect Molecular Biology, 21, 61–77. 22032702 10.1111/j.1365-2583.2011.01113.x PMC3540788

[imb12575-bib-0050] Saavedra‐Rodriguez, K. , Urdaneta‐Marquez, L. , Rajatileka, S. , Moulton, M. , Flores, A.E. , Fernandez‐Salas, I. et al. (2007) A mutation in the voltage‐gated sodium channel gene associated with pyrethroid resistance in Latin American *Aedes aegypti* . Insect Molecular Biology, 16, 785–798. 18093007 10.1111/j.1365-2583.2007.00774.x

[imb12575-bib-0051] Stevenson, B.J. , Pignatelli, P. , Nikou, D. and Paine, M.J. (2012) Pinpointing P450s associated with pyrethroid metabolism in the dengue vector, *Aedes aegypti*: developing new tools to combat insecticide resistance. PLoS Neglected Tropical Diseases, 6, e1595. 22479665 10.1371/journal.pntd.0001595 PMC3313934

[imb12575-bib-0052] Strode, C. , De Melo‐Santos, M. , Magalhaes, T. , Araujo, A. and Ayres, C. (2012) Expression profile of genes during resistance reversal in a temephos selected strain of the dengue vector, *Aedes aegypti* . PLoS One, 7, e39439. 22870187 10.1371/journal.pone.0039439 PMC3411583

[imb12575-bib-0053] Strode, C. , Wondji, C.S. , David, J.P. , Hawkes, N.J. , Lumjuan, N. , Nelson, D.R. et al. (2008) Genomic analysis of detoxification genes in the mosquito *Aedes aegypti* . Insect Biochemistry and Molecular Biology, 38, 113–123. 18070670 10.1016/j.ibmb.2007.09.007

[imb12575-bib-0058] Vera-Maloof, F.Z. , Saavedra-Rodriguez, K. , Elizondo-Quiroga, A.E. , Lozano-Fuentes, S. and Black Iv, W.C. (2014) Coevolution of the Ile1,016 and Cys1,534 mutations in the voltage gated sodium channel gene of *Aedes aegypti* in Mexico. PLoS Neglected Tropical Diseases, 9(12), PMC4684211. 10.1371/journal.pntd.0004263 PMC4684211 26658798

[imb12575-bib-0054] Wu, T.D. and Nacu, S. (2010) Fast and SNP‐tolerant detection of complex variants and splicing in short reads. Bioinformatics, 26, 873–881. 20147302 10.1093/bioinformatics/btq057 PMC2844994

[imb12575-bib-0055] Yan, G. , Chadee, D.D. and Severson, D.W. (1998) Evidence for genetic hitchhiking effect associated with insecticide resistance in *Aedes aegypti* . Genetics, 148, 793–800. 9504925 10.1093/genetics/148.2.793 PMC1459830

[imb12575-bib-0056] Yan, L. , Yang, P. , Jiang, F. , Cui, N. , Ma, E. , Qiao, C. et al. (2012) Transcriptomic and phylogenetic analysis of *Culex pipiens quinquefasciatus* for three detoxification gene families. BMC Genomics, 13, 609. 23140097 10.1186/1471-2164-13-609 PMC3505183

[imb12575-bib-0057] Zalucki, M.P. and Furlong, M.J. (2017) Behavior as a mechanism of insecticide resistance: evaluation of the evidence. Current Opinion in Insect Science, 21, 19–25. 28822484 10.1016/j.cois.2017.05.006

