# Supplementary figures and images for: Exome‐wide association of deltamethrin resistance in Aedes aegypti from Mexico
Source: Insect Mol Biol. 2019 Mar 13;28(5):591–604. doi: 10.1111/imb.12575 (PMC6766855; doi:10.1111/imb.12575)

## Chromosome 1

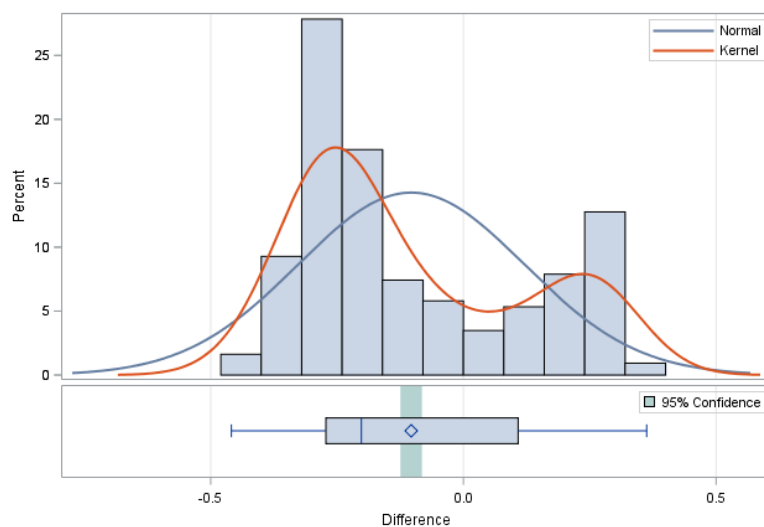

## Chromosome 2

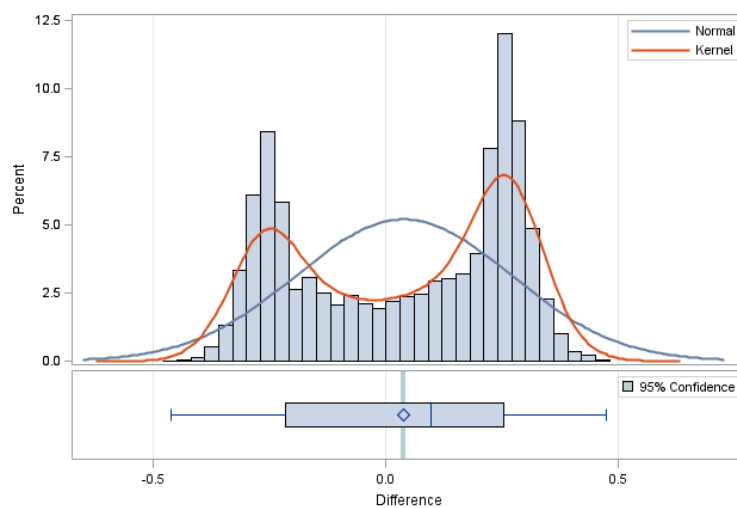

## Chromosome 3

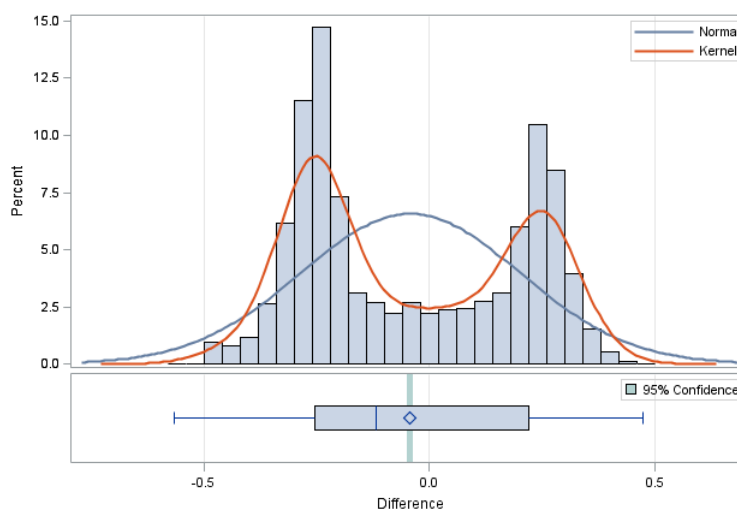

Supplement: Supplementary file 1 — Figure S1. Distribution of the differences in heterozygosity in knockdown resistant versus susceptible mosquitoes (Hexp alive (HETA) Hexp dead (HETD)) at each significant SNP in chromosomes 1, 2 and 3. [file IMB-28-591-s001.pdf]
