# Supplementary material for: Exome‐wide association of deltamethrin resistance in Aedes aegypti from Mexico
Source: Insect Mol Biol. 2019 Mar 13;28(5):591–604. doi: 10.1111/imb.12575 (PMC6766855; doi:10.1111/imb.12575)
Supplement: Supplementary file 3 — Figure S3. List of significant detoxification genes in our study and the expression that has been reported in previous studies. Upregulated genes are highlighted in orange, downregulated are highlighted in blue, downregulated or upregulated in the same study are highlighted in grey. A number represents the number of polymorphisms found at a particular SNP in that gene. [file IMB-28-591-s003.pdf]

			Study																					
Gene	LOC	VBN	1	2	3	4	5	6	7	8	9	10	11	12	13	14	15	16	17	18	19	20	21	22
CCE																								
CCEAE1O	LOC5564576	AAEL004341																						
CCEAE1A	LOC5578161	AAEL005113																						
CCEunk5O	LOC5563677	AAEL000546																						
CCEJHE2O	LOC5564562	AAEL004323																						
CCEglt1G	LOC5567206	AAEL000889																						
CCEglt1K	LOC5567437	AAEL019933																						
CCEunk1O	LOC5563680	AAEL000530																						
CCEAE3O	LOC5575614	AAEL011944																						
CCEAE3A	LOC5566025	AAEL023844																					2	
CCEAE4A	LOC5566008	AAEL019678																					8	
CCEAE6A	LOC5566034	AAEL015264																						
CCEAE2B	LOC23687974	AAEL017553																						
CCEAE3B	LOC5574471	AAEL002385																						
CCEAE4B	LOC5574470	AAEL002378																						
CCEAE5B	LOC5574469	AAEL002391																						
CCEAE1D	LOC23687492	AAEL015578																						
CCEAE2D	LOC5573294	AAEL010389		sign													1							
CYP																								
CYP12F5	LOC5573118	AAEL001960															1							
CYP303A1	LOC5575902	AAEL012144																						
CYP325M2	LOC5576781	AAEL012769																						
CYP325Q1	LOC5567399	AAEL006044																						
CYP325T1	LOC5575193	AAEL000320																						
CYP4AR2	LOC5572943	AAEL010154																						
CYP4D37	LOC5569663	AAEL007795																						
CYP4D38	LOC5569664	AAEL007807																						
CYP4H33	LOC5578647	AAEL013798																						
CYP4J13	LOC5565337	AAEL013555																						
CYP4J14	LOC5578182	AAEL013554															1							
CYP6AK1	LOC5565729	AAEL004941																						
CYP6AL1	LOC5571194	AAEL008889																						
CYP6N14	LOC5571533	AAEL009133																						
CYP6S3	LOC5571532	AAEL009120																					2	
CYP6Z7	LOC5571543	AAEL009130																						
CYP302A1	LOC5574851	AAEL011463																						
CYP305A6	LOC5573424	AAEL026706																					1	
CYP325G2	LOC5576784	AAEL012766																						
CYP325G3	LOC5576790	AAEL012772																						

CYP325K3	LOC5567036	AAEL005788																						
CYP325L1	LOC5575359	AAEL011770																						
CYP325M1	LOC5576777	AAEL012773																						
CYP325M3	LOC5576782	AAEL012765																						
CYP325M4	LOC5575362	AAEL020553																						
CYP325N1	LOC5576778	AAEL012770																						
CYP325N2	LOC5576783	AAEL012762																						
CYP325P1	LOC5575192	AAEL000340																						
CYP325Q2	LOC5579337	AAEL006058																						
CYP325T2	LOC5576780	AAEL012761																						
CYP325Z	LOC5567665	AAEL019960																				1	
CYP49A1	LOC5570873	AAEL008638																						
CYP4C38	LOC5576029	AAEL012266														1							
CYP4D24	LOC5569666	AAEL007815																						
CYP4D39	LOC5569665	AAEL007808																						
CYP4H32	LOC5569660	AAEL007812		sign																				
CYP4J15	LOC5578183	AAEL013556																				1	
CYP4J16	LOC5579145	AAEL014019																						
CYP6AG5	LOC5568652	AAEL006984																						
CYP6AG6	LOC5568653	AAEL006992																						
CYP6AG7	LOC5568654	AAEL006989																						
CYP6M6	LOC5571538	AAEL009128														3						1	
CYP6N11	LOC5571527	AAEL009138																				1	
CYP6N12	LOC5571530	AAEL009124														1							
CYP6N13	LOC5571525	AAEL009137																						
CYP6N15	LOC5571534	AAEL009122																						
CYP6N16	LOC5572937	AAEL010151																						
CYP6N6	LOC5571529	AAEL009126														1							
CYP6Y3	LOC5571537	AAEL009132														1							
CYP6Z9	LOC5571545	AAEL009129														2							
CYP9J15	LOC5579927	AAEL006795																						
CYP9J16	LOC5579928	AAEL006815																						
CYP9J18v2	LOC5564760	AAEL014618																						
CYP9J22v2	LOC5564749	AAEL014619																						
CYP9J29	LOC5564759	AAEL014610		sign																				
CYP9J30	LOC5564761	AAEL014603																						
CYP9J32	LOC5571142	AAEL019603		sign																				
CYP9J8	LOC5564754	AAEL019504																						
CYP6M11	LOC5571541	AAEL009127																						
CYP9M4	LOC5569821	AAEL001320																						
CYP9M5	LOC5569822	AAEL025530																						

CYP9M7	LOC5569824	AAEL001292																						
CYP9M9	LOC5572442	AAEL001807																						
GST																								
GSTD2	LOC5568355	AAEL001078																						
GSTX1	LOC5563651	AAEL000092																					1	
GSTO1	LOC23687506	AAEL017085																						
Redox																								
HPX1	LOC5567514	AAEL006014																						
HPX6	LOC5575608	AAEL011941																						
HPX8C	LOC5564685	AAEL004386																						
MNSOD2	LOC5566004	AAEL005108																						
aldo-keto re	LOC5565865	AAEL015002																						
ALDOX 1037	LOC5573297	AAEL010370																						
ALDOX 1038	LOC5573275	AAEL010380																						
ALDOX 1038	LOC5573295	AAEL010384																						
ALDOX 1036	LOC5573281	AAEL010367																						
ALDOX 1037	LOC5573298	AAEL026787																						
ALDOX 1038	LOC5573296	AAEL010382																						
ALDOX 1039	LOC5573283	AAEL024680																					1	
aldo-keto re	LOC5564125	AAEL004086																						
aldo-keto re	LOC5564134	AAEL004088																						
dihydrolipo	LOC5568522	AAEL006928																						
DUOX	LOC5569379	AAEL007563																						

Up	Down	Up/Down

Supplementary Table 6. List of significant detoxification genes in our study and the expression that has been reported in previous studies. Upregulated genes are highlighted in orange, downregulated are highlighted in blue, downregulated or upregulated in the same study are highlighted in grey. If a numerical is shown, it represents the number of polymorphisms found at a particular SNP in that gene, sign= significantly associated with resistance.

[1]Poupardin et al 2008; [2]Saavedra-Rodriguez et al 2008; [3]Strode et al 2008;[4]Poupardin et al 2009; [5]Riaz et al 2009;[6]Marcombe et al 2009; [7]David et al 2010; [8]Saavedra-Rodriguez 2011; [9]Bingham et al 2011; [10]Bariami et al 2012; [11]Poupardin et al 2012; [12]Strode et al 2012; [13]Marcombe et al 2012; [14]Grisales et al 2013; [15]David et al 2014; [16]Saavedra-Rodriguez et al 2013; [17]Poupardin et al 2013; [18]Kasai et al 2014; [19]Reid et al 2014; [20]Dusofur et al 2015; [21]Faucon et al 2015; [22]Estep et al 2017.
