# Supplementary material for: Exome‐wide association of deltamethrin resistance in Aedes aegypti from Mexico
Source: Insect Mol Biol. 2019 Mar 13;28(5):591–604. doi: 10.1111/imb.12575 (PMC6766855; doi:10.1111/imb.12575)
Supplement: Supplementary file 4 — Table S1. Number of reads aligned AaeL5 across chromosome 1, 2 and 3 for each replicated libraries. The number of common reads between replicates and among groups (knockdown resistant vs susceptible) is shown. [file IMB-28-591-s004.docx]

**Supplementary Table 1**. Number of reads in deltamethrin resistant and susceptible libraries of *Ae. aegypti*. The common reads are shown between the two biological replicates and the common SNPs between resistant and susceptible groups. Polymorphic SNPs were used for further analyses.

| Phenotype | Replicate 1 | | Replicate 2 | Common reads between replicates |
| --- | --- | --- | --- | --- |
|  |  | |  |  |
| **Knockdown resistant** |  | |  |  |
| Chromosome 1 | 392,543 | | 1,177,242 | 335,304 |
| Chromosome 2 | 5,659,170 | | 7,871,974 | 5,476,085 |
| Chromosome 3 | 3,369,072 | | 5,525,437 | 3,186,806 |
| Total | 9,420,785 | | 14,574,653 | 8,998,195 |
| **Susceptible** |  | |  |  |
| Chromosome 1 | 536,753 | | 633,192 | 300,557 |
| Chromosome 2 | 6,175,614 | | 6,615,092 | 5,357,833 |
| Chromosome 3 | 3,793,462 | | 4,194,135 | 2,994,424 |
| Total | 10,505,829 | | 11,442,419 | 8,652,814 |
| **Resistant vs Susceptible** | | Common  sites | Monomorphic sites | Polymorphic  sites |
| Chromosome 1 | 174,821 | | 163,597 | 11,224 |
| Chromosome 2 | 4,563,089 | | 4,257,562 | 305,527 |
| Chromosome 3 | 2,317,151 | | 2,179,522 | 137,629 |
| Total | 7,055,061 | | 6,600,681 | 454,380 |
